# Phytoconstituents from *Alpinia purpurata* and their *in vitro* inhibitory activity against *Mycobacterium tuberculosis*

**DOI:** 10.4103/0973-1296.71785

**Published:** 2010

**Authors:** Oliver B. Villaflores, Allan Patrick G. Macabeo, Dietmar Gehle, Karsten Krohn, Scott G. Franzblau, Alicia M. Aguinaldo

**Affiliations:** *Phytochemistry Laboratory, Research Center for the Natural Sciences, Thomas Aquinas Research Complex, University of Santo Tomas, España, Manila 1008, Philippines*; 1 *University of Paderborn, Department of Chemistry, Warburgerstrasse 100, 33098 Paderborn, Germany*; 2 *Institute for TB Research, College of Pharmacy, MC 964 Rm 412, University of Illinois at Chicago, 833 S. Wood St., Chicago, IL 60612-7231 USA*

**Keywords:** *Alpinia purpurata*, fatty alcohols, kumatakenin, *Mycobacterium tuberculosis*, sitosteryl glycosides

## Abstract

*Alpinia purpurata* or red ginger was studied for its phytochemical constituents as part of our growing interest on Philippine Zingiberaceae plants that may exhibit antimycobacterial activity. The hexane and dichloromethane subextracts of the leaves were fractionated and purified using silica gel chromatography to afford a mixture of C_28_–C_32_ fatty alcohols, a 3-methoxyflavone and two steroidal glycosides. The two latter metabolites were spectroscopically identified as kumatakenin (1), sitosteryl-3-O-6-palmitoyl-β-D-glucoside (2) and b-sitosteryl galactoside (3) using ultraviolet (UV), infrared (IR), electron impact mass spectrometer (EIMS) and nuclear magnetic resonance (NMR) experiments, and by comparison with literature data. This study demonstrates for the first time the isolation of these constituents from **A. purpurata**. In addition to the purported anti-inflammatory activity, its phytomedicinal potential to treat tuberculosis is also described.

## INTRODUCTION

Several species of the genus Alpinia have been reported to exhibit fungicidal, antioxidant and bactericidal properties.[1,2] Alpinia purpurata (Vieill.) K. Schum (Family: Zingiberaceae) is locally known in the Philippines as “luyang pula” or red ginger, and is a native to the Pacific.[3] Studies on its chemical constituents revealed the presence of α-pinene, β-pinene,[4] 1,8-cineole, (*E*)-methylcinnamate,[5] 6-shogaol, 8-gingerol, 6-gingerol, 10-gingerol, 10-shogaol and 4-shogaol.[6] A US patent reported that its total anthocyanidin, shogaol and gingerol content shows promise in the treatment of inflammatory diseases such as arthritis.[6,7]

**Figure 1:**
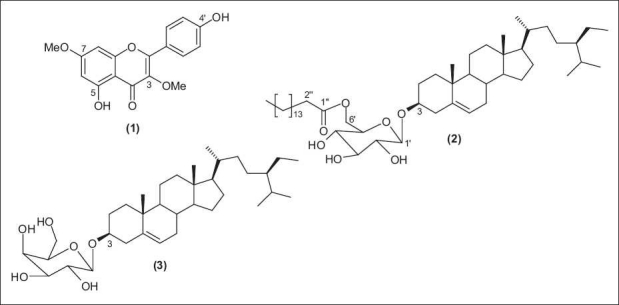
Flavone and sitosteryl glycosides from **A. purpurata**

With limited literature available as to the phytochemistry and biological activity of **A. purpurata**, and with the growing interest in Philippine Zingiberaceae species that inhibit the growth of *Mycobacterium tuberculosis* H_37_ Rv,[8,9] we embarked on further exploration on the isolation and identification of secondary metabolites from this *Alpinia* species. In addition to the fatty alcohol mixture, we report in this paper the chromatographic purification and spectroscopic identification of a flavone and two sitosteryl glycosides, namely, kumatakenin (1), sitosteryl-3-O-6-palmitoyl-β-d-glucoside (2) and β-sitosteryl galactoside (3) from **A. purpurata** [Figure 1]. The inhibitory activity against *M. tuberculosis* H37Rv of the extracts, fractions and the purified compounds is also presented.

## MATERIALS AND METHODS

### General

The leaves of **A. purpurata** were collected from Los Baños, Laguna, during February 2004. Herbarium specimens (USTH 4717) were kept at the Botany Laboratory of the Research Center for the Natural Sciences, Thomas Aquinas Research Complex, University of Santo Tomas, Manila, Philippines.

Electron impact mass spectral (EIMS) analysis was carried out with a JEOL D-300 FD mass spectrometer, using *m*-nitrobenzyl alcohol/CHCl_3_ as carrier at 60°C. Proton (^1^H) and ^13^C nuclear magnetic resonance (NMR) measurements were recorded with a JEOL GX 400 MHz NMR spectrometer using CDCl_3_ (δ 7.26 for ^1^H, δ 77.0 for ^13^C) as internal reference.

### Extraction and isolation

The air-dried leaves of **A. purpurata** (1.7 kg) were extracted with ethanol to give a crude extract (378 g) that was partitioned according to increasing polarity using *n*-hexane, dichloromethane and *n*-butanol.

The hexane extract (53.4 g) was subjected to vacuum liquid chromatography (VLC) by gradient elution (20% increments) using hexane/ethyl acetate to give 11 fractions. Fraction 2 (8.4 g) was purified by gravity column chromatography (GCC) and gradient elution (5% increments) with hexane/chloroform and chloroform/acetone, to give four fractions. Sub-fraction 2 was further purified and it gave 19 fractions. A solid in the eighth fraction was recrystallized in acetone to afford a white amorphous solid (10 mg) of a fatty alcohol mixture.

The dichloromethane extract (2.5 g) was subjected to VLC (Si gel HF_254_ Merck Art. 1.07739) by gradient elution (20% increments), using chloroform, chloroform-acetone, acetone and acetone-methanol to furnish nine fractions.

After evaporation of fraction 3, yellow needle-like crystals appeared and were recrystallized in acetone to give **1** (13 mg). UV spectral analysis was also done on compound **1** using various shift reagents to determine the aromatic substitutions of the compound. Fraction four (458 mg) was gravity column chromatographed (Si gel 60 Merck Art. 1.07734, 25 mm I.D.) by gradient elution with benzene-acetone (5:1), (5:2), (5:3), neat acetone, acetone-methanol (1:1) and neat methanol to give nine fractions. Sub-fraction eight gave an amorphous white powder that was recrystallized in acetone to give **2** (8.4 mg). Concentration and recrystallization of sub-fraction 6 in methanol afforded **3** (4.1 mg) as white flakes.

**Fatty alcohols**: ^1^H NMR (500 MHz, CDCl_3_) 3.57 (t, J 6.6 Hz, H-1), 1.49 (m, H-2), 1.18 (br s), 0.81 (t, J 7 Hz). ^13^C NMR (DEPT-135, 125 MHz, CDCl_3_) 63.1 (C-1), 32.8 (C-2), 31.9 (C-3), 29.7, 25.7(C-3), 22.6, 14.1. LR-EIMS m/z: 392.5 (C_28_H_57_OH-H_2_O)^+^, 420.5 (C_30_H_61_OH-H_2_O)^+^, 448(C_32_H_65_OH-H_2_O)^+^.

*Kumatakenin* **(1)** : yellow needles (13 mg), m.p. 248–249°C. ^1^H NMR (500 MHz, CDCl_3_/CD_3_ OD) 3.69 (3H, s, 3-OMe), 3.78 (3H, s, 7-OMe), 6.24 (1H, d, J 2 Hz, H-6), 6.37 (1H, d, J 2 Hz, H-8), 6.82 (2H, d, J 9 Hz, H-3’, H-5’), 7.89 (2H, d, J 9 Hz, H-2’, H-6’. ^13^C NMR (DEPT-135, 125 MHz, CDCl_3_/CD_3_ OD) 156.7 (C-2), 138.4 (C-3), 178.7 (C-4), 161.3 (C-5), 97.8 (C-6), 165.4 (C-7), 92.2 (C-8), 156.8 (C-9), 105.8 (C-10), 121.3 (C-1’), 130.2 (C-2’), 115.5 (C-3’, C-5’), 159.9 (C-4’), 130.2 (C-2’, C-6’), 59.9 (3-OMe), 55.6 (7-OMe). LR-EIMS *m/z*: 314.1 (M^+^), 271.1, 256.2, 167.0, 149.0, 97.1, 57.1, 43.0.

*Sitosteryl-3-O-6-palmitoyl-β-D-glucoside* **(2)** : white amorphous powder (8.4 mg). ^1^H NMR (500 MHz, CDCl_3_/CD_3_OD) *aglycone* 3.58 (1H, m, H-3), 5.36 (1H, m, H-6), 0.70 (3H, s, 18-CH_3_), 1.03 (3H, s, 19-CH_3_), 0.94 (3H, d, J 6.5 Hz, 21-CH_3_), 0.85 (3H, d, J 6.8 Hz, 26-CH_3_), 0.84 (3H, d, J 6.8 Hz, 27-CH_3_), 0.90 (3H, t, J 6.9 Hz, 29-CH_3_); sugar 4.38 (1H, d, J 7.7 Hz, H-1’), 3.34 (1H, m, H-2’), 3.58 (1H, m, H-3’), 3.34 (1H, m, H-4’), 3.48 (1H, ddd, J 2 Hz, 5 Hz, 10 Hz, H-5’), 4.29 (1H, dd, J 2 Hz, 12 Hz, H-6’a), 4.48 (1H, dd, J 5 Hz, 12 Hz, H-6’b); *fatty acid* 2.37 (2H, t, J 7.5 Hz, H-2”), 1.72 (2H, m, H3”), 1.28 (24H, broad s, H-4”-15”), 0.87 (3H, t, J 7 Hz, H-16”). ^13^C NMR (DEPT-135, 125 MHz, CDCl_3_/CD_3_OD); *aglycone* 37.3 (C-1), 31.9 (C-2), 79.6 (C-3), 38.9 (C-4), 140.3 (C-5), 122.2 (C-6), 31.9 (C-7), 31.9 (C-8), 50.2 (C-9), 36.7 (C-10), 21.2 (C-11), 39.8 (C-12), 42.2 (C-13), 56.8 (C-14), 25.0 (C-15), 28.2 (C-16), 56.1 (C-17), 11.9 (C-18), 19.4 (C-19), 36.2 (C-20), 19.0 (C-21), 34.0 (C-22), 26.1 (C-23), 45.9 (C-24), 29.2 (C-25), 18.8 (C-26), 19.8 (C-27), 23.1 (C-28), 12.0 (C-29); *sugar* 101.2 (C-1’), 73.6 (C-2’), 76.0 (C-3’), 70.1 (C-4’), 74.0 (C-5’), 63.2 (C-6’); *fatty acid* 174.7 (C=O), 34.2 (C-2”), 24.3 (C-3”), 28.9-29.7 (C-4”–14”), 22.6 (C-15”), 14.1 (C-16”). LR-EIMS *m/z*: 414.7 (aglycone), 396.4, 381.3, 368.4, 329.3, 284.3, 256.3, 241.2, 239.2, 227.0, 213.2.

*β-sitosteryl galactoside* **(3)**: white flakes (4.1 mg). ^1^H NMR (500 MHz, CDCl_3_ /CD_3_ OD); *aglycone* 3.47 (1H, m, H-3), 5.25 (1H, m, H-6), 0.57 (3H, s, 18-CH_3_), 0.90 (3H, s, 19-CH_3_), 0.73 (3H, d, J 7 Hz, 21-CH_3_), 0.70 (3H, d, J 7 Hz, 26-CH_3_), 0.82 (3H, d, J 7.8 Hz, 27-CH_3_), 0.74 (3H, t, J 7.8 Hz, 29-CH_3_); *sugar* 4.29 (1H, d, J 8 Hz, H-1’), 3.12 (1H, dd, J 8 Hz, 9 Hz, H-2’), 3.32 (1H, dd, J 1 Hz, 9 Hz, H-3’), 3.32 (1H, dd, J 1 Hz 7 Hz, H-4’), 3.18 (1H, m, H-5’), 3.63 (1H, dd, J 5 Hz, 12 Hz, H-6’a), 3.73 (1H, dd, J 3 Hz, 12 Hz, H-6’b). ^13^C NMR (DEPT-135, 125 MHz, CDCl_3_ /CD_3_ OD); *aglycone* 37.2 (C-1), 29.6 (C-2), 79.0 (C-3), 38.6 (C-4), 140.2 (C-5), 122.0 (C-6), 31.0 (C-7), 31.0 (C-8), 50.1 (C-9), 36.6 (C-10), 21.0 (C-11), 39.7 (C-12), 42.2 (C-13), 56.6 (C-14), 24.1 (C-15), 28.1 (C-16), 56.0 (C-17), 11.7 (C-18), 19.5 (C-19), 36.0 (C-20), 19.1 (C-21), 33.9 (C-22), 26.0 (C-23), 45.7 (C-24), 29.4 (C-25), 18.6 (C-26), 18.8 (C-27), 22.9 (C-28), 11.8 (C-29); *sugar* 101.1 (C-1’), 73.4 (C-2’), 76.3 (C-3’), 70.2 (C-4’), 75.6 (C-5’), 61.7 (C-6’). LR-EIMS *m/z* 414.4 (*aglycone*), 397.4, 396.4, 381.4, 329.3, 288.3, 255.2, 213.2.

Assignments were made by comparison with published data and confirmed by HMQC/COSY experiments.

### Screening for antituberculosis activity

Microplate Alamar Blue Assay (MABA), as described in the protocol of Collins and Franzblau,[10] was used to test the anti-TB susceptibility of the extracts, fractions and purified compounds. *M. tuberculosis* H_37_ Rv (ATCC 27294; American Type Culture Collection, Rockville, MD, USA) was grown at 37°C on a rotary shaker in Middlebrook 7H9 broth supplemented with 2% glycerol and 0.05% v/v Tween 80, until the culture density reached an optical density of 0.45–0.55 at 550 nm. Bacteria were pelleted, washed twice, resuspended in Dulbecco’s phosphate-buffered saline, then filtered (8 μm) and aliquots frozen at −80°C. After a night, the stocks were thawed, sonicated and successively diluted to get the colony forming units (CFU). Rifampin was obtained from Sigma and stock solutions were made in accordance with the manufacturer’s instructions. The assay was performed in black, clear-bottomed, 96-well microplates (Black view plates, Packard Instrument company, Meriden, CT, USA) in order to reduce background fluorescence. Initial drug dilutions were prepared in either dimethyl sulfoxide or distilled ionized water, and subsequent twofold dilutions were performed in 0.1 ml of 7H9GC (no Tween 80) in the microplates. BACTEC 12B-passaged inocula were initially diluted 1:2 in 7H9GC and 0.1 ml was placed onto the wells. Frozen inocula were diluted 1:20 in BACTEC 12B medium, followed by a 1:50 dilution in 7H9GC. Wells containing drug were used to monitor autofluorescence of compounds. Additional control wells consisted of bacteria only (B) and medium only (M). Plates were incubated at 37°C. On day 4 of incubation were added 20 μl of 10× Alamar Blue solution (Alamar Biosciences/Accumed, Westlake, OH, USA) and 12.5 μl of 20% Tween 80 to one B well and one M well, and plates were reincubated at 37°C. Color changes from blue to pink were monitored in the wells at 12 and 24 h and for a measurement reading of ≥50,000 fluorescence units (FU). Cytofluor II microplate flurometer (PerSeptive Biosystems, Framingham, MA, USA) in bottom-reading setting at 530 nm for excitation and 590 nm for emission was used in fluorescence measurement. In case a pink color is observed with B wells after 24 h, the colorimetric reagent is added to the entire plate. If a blue color persists in the well or a reading of ≤50,000 FU is obtained, additional wells containing bacteria and medium were tested daily until a change in color is observed. At this point, reagents were added to other remaining wells. At 37°C, the plates were incubated and the results were noted 24 h post-reagent addition. Visual minimum inhibitory concentrations (MICs) were defined as the lowest concentration of drug that resisted a color change. A background subtraction was performed on all wells with a mean triplicate M wells for fluorimetric MICs. Percent inhibition was defined as 1 - (test well FU/mean FU of triplicate B wells) × 100. The lowest drug concentration exhibiting an inhibition of ≥90% was assigned as the MIC.

## RESULTS AND DISCUSSION

The MABA assay[10] results of the crude ethanolic extract of the various parts of **A. purpurata** have shown the leaf extract to possess the highest activity, followed by the rhizome and flower extracts. Among the sub-extracts, the dichloromethane (DCM) sub-extract exhibited the highest activity, followed by hexane and *n*-butanol sub-extracts. All fractions obtained from the hexane and DCM sub-extracts showed low to moderate activity [Table 1].

**Table 1:** Percent inhibitory activity of the extracts, sub-extracts and fractions of **A. purpurata** at 64 μg/ml against *M. tuberculosis* H_37_Rv

Sample	%Inhibition
Crude ethanolic extracts	
Flowers	30
Leaves	62
Rhizome	34
Leaf sub-extracts	
Hexane	64
DCM	72
*n*-Butanol	35
Hexane fractions (APH)	
APH 1	47
APH 2	38
APH 3	55
APH 4	48
APH 5	61
APH 6	74
APH 7	73
APH 8	70
APH 9	74
APH 10	46
APH 11	23
DCM fractions (APD)	
APD 1	65
APD 2	58
APD 3	68
APD 4	60
APD 5	69
APD 6	66
APD 7	46
APD 8	50
APD 9	64

Control: rifampin, 99% at 0.18 μg/ml

Further chromatographic work-up was undertaken on fraction two of the hexane sub-extract, which afforded a white amorphous solid after crystallization. This compound was distinctly identified by the ^1^H-NMR and ^13^C-NMR spectra to be a fatty alcohol, but the LR-EIMS spectrum otherwise showed it to be a mixture of fatty alcohols. By careful analysis of the *m/z* values in the mass spectrum, it could be claimed that it is composed of montanyl alcohol (C28:0), melissyl alcohol (C30:0, major component) and domelissyl alcohol (C32:0), based on several characteristic peaks due to fragment ions of [M^+^-H_2_O].[11]

The dichloromethane extract was likewise subjected for further investigation owing to its interesting phytochemical profile. Nine fractions were obtained after VLC from which fractions three and four yielded three solid compounds **1-3**.

Compound **1**, a yellow crystalline substance (13.0 mg), was purified from fraction three after recrystallization. It was found to be a flavonoid after treating its thin layer chromatograms with FeCl_3_-K_4_ Fe(CN)_6_ and 10% SbCl_3_ in chloroform, as shown by a blue-green spot and red-orange fluorescence, respectively, under UV (530 nm).[12] In addition, major absorptions at 269 nm (band I) and 352 nm (band II) and a weak shoulder at 303 nm, which are typical for flavones, were observed in its UV spectrum.[13,14] The structure, and degree and pattern of oxygenation in the flavonoid structure were examined by studying the effect of several wavelength shift reagents, NaOMe, NaOAc, NaOAc-1% aq. H_3_ BO_3_, AlCl_3_ and AlCl_3_ -HCl in the UV spectral region of 1. With NaOMe, a 44-nm bathochromic shift and a significant increase in absorbance intensity were noted for band II. This indicates the presence of a C-4’ hydroxyl group. Treatment with NaOAc gave no observable change in the spectrum, which shows that an alkoxy substituent is present in the C-7 position of the flavone nucleus. Addition of 1% boric acid produced no change in the spectrum, which is symptomatic of the absence of ortho-hydroxyphenolic functionalities. This was also substantiated by the result of adding 0.1 M HCl/AlCl_3_ in a separate experiment. The appearance of four absorption peaks (278, 304, 352 and 399 nm) after the complexation of AlCl_3_ with **1** is a clear indication that a 5-hydroxyl moiety is present.[14]

**Figure 2:**
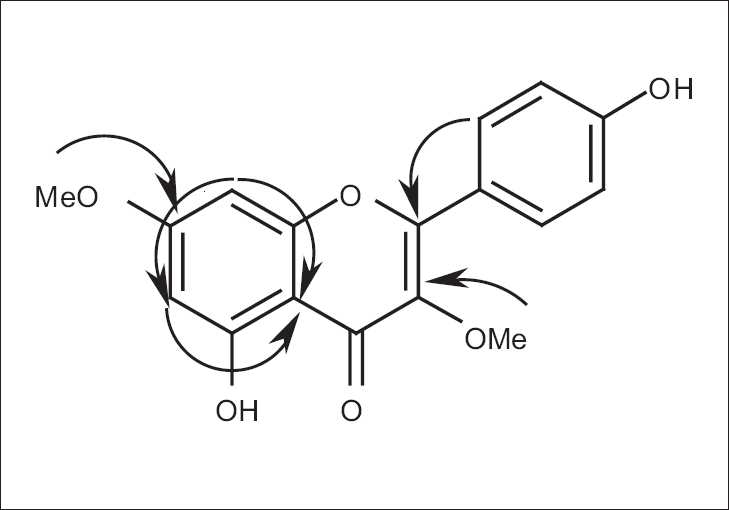
Key ^1^H-^13^C HMBC correlations in 1

The IR spectrum of 1 showed the presence of an enone (1665 cm^-1^) and phenolic OH’s (3243 cm^-1^). The base peak at *m/z* 314 in the LR-EIMS mass spectrum was designated the molecular-ion peak. In the 500-MHz ^1^H NMR spectrum, two sets of AA´BB´-protons belonging to a para-substituted benzenoid moiety, two methoxy protons and meta-coupled protons were noted. In the proton-decoupled ^13^C and DEPT-135 NMR spectrum, a total of 18 carbon atoms were accounted for 1, from which a conjugated ketone, six oxygenated olefinic/aromatic carbons, six aromatic methines and two methoxy carbons were deduced. The gross structure of the compound which is analogous to kumatakenin[15,16] was elucidated via an HMBC experiment. Key ^1^H-^13^C correlations are shown in Figure 2.

It is noteworthy to report the isolation of kumatakenin (**1**) from **A. purpurata**. This rare ethyl ether flavonol was first isolated from the seeds of *Alpinia japonica*[17] and *Alpinia kumatake*.[18] Hence, the identification of **1** strengthens the chemical link of **A. purpurata** with the other species of *Alpinia*.

**Figure 3:**
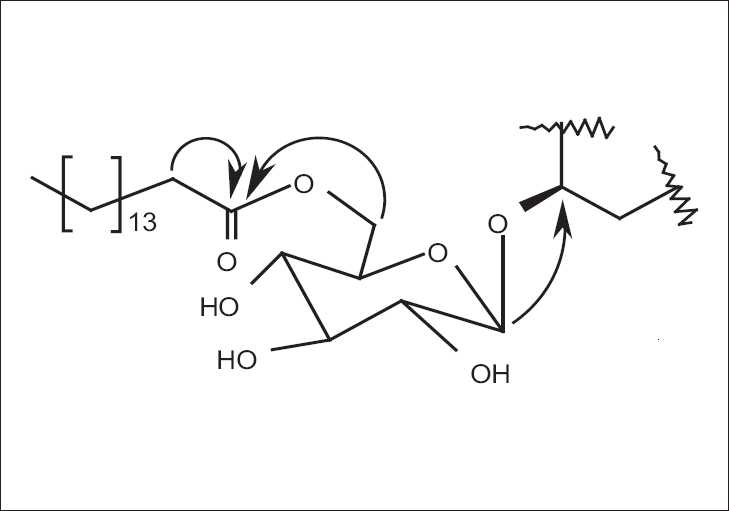
Key ^1^H-^13^C HMBC Correlations in 2

Compound **2** was obtained as a white amorphous solid (8.4 mg). Thin layer chromatograms of the isolate sprayed with Lieberman*n*-burchard and Molisch reagents[12] suggested a steroidal glycoside structure. The IR spectrum showed the presence of hydroxyl (3439 cm^-1^) and ester (1738 cm^-1^) functionalities. The molecular ion peak was not observed in the LR-EIMS spectrum. Instead, fragment ions corresponding to sitosterol (*m/z*414, C_29_H_50_O) and a palmitoxy group (*m/z*256, C_16_H_31_O_2_) were noted. Signals characteristic of sitosterol resonances, i.e., C-6 olefinic proton (δ 5.36), methyls associated with the cholestane skeleton (δ 0.69–0.79), glucose (δ 4.34–4.38) and a palmitoyl group (δ 0.84, 1.26, 2.34) were evident in the 500-MHz ^1^H NMR of 2. A total of 51 carbon atoms, i.e., 7 CH_3_, 25 aliphatic CH_2_, 7 aliphatic CH, 2 aliphatic quaternary C, 1 oxygenated CH_2_, 6 oxygenated CH and one each olefinic CH and C, were found in the 125 MHz ^13^C NMR spectrum. HMBC correlations, which were instrumental in finding the correct identity of 2 as sitosteryl-3-O-6-palmitoyl-β-d-glucoside, are shown in Figure 3. The NMR values of 2 are well in agreement with those reported for sitosteryl-3-O-6-palmitoyl-β-d-glucoside by Pei-Wu *et al*.,[19] Gomes *et al*.,[20] and Shaiq Ali *et al*.[21]

Metabolite **3** was obtained as white crystalline flakes (4.1 mg). The partial identity of *3* was revealed to be a steroidal glycoside as in **2** using the same phytochemical experiments. Only the occurrence of hydroxyl functionalities was inferred this time from the IR spectrum. The presence of a sitosterol fragment was ascertained in the LR-EIMS spectrum, which was also verified by the ^1^H and ^13^C NMR spectra. The *sugar* moiety was deduced to be galactose upon comparison of the ^1^H-NMR and ^13^C-NMR values with those in literature.[22–24] Hence, the identity of **3** was established.

Sitosteryl glycosides **2** and **3** have been isolated from other several plant sources.[19,21] These metabolites are also present in plant species belonging to the family Zingiberaceae.[25]

The fatty alcohols showed an MIC value of 64 μg/ml and proved most active compared to the flavonoid kumatakenin and the steroidal glycosides previously reported to have MIC values >128 μg/ml. Long chain alcohols show growth inhibitory activity to Gram-positive organisms including *Staphylococcus aureus* and *Propionibacterium acnes*.[26] Moreover, a study done by Togashi and co-workers in 2007 further supported the antibacterial activity of long chain aliphatic alcohols that had bactericidal activity and membrane-damaging activity on *S. aureus*. Experimental results indicate that the antibacterial activity of long chain alcohols is mediated by damage to cell membranes, which allows leakage of K^+^ ions, with subsequent reactions that induce further leakage.[27] Thus, the ability of the isolated fatty alcohols to inhibit the growth of *M. tuberculosis* H_37_Rv gives further credence on the antibacterial activity of long chain alcohols, particularly those bearing more than 20 carbon chains.

The activity of kumatakenin confirms the study by Murillo *et al*.[28] on its action against *M. tuberculosis* H_37_ Rv. This compound has antiviral activity against HIV,[29] the virus that aggravates the problem on tuberculosis (TB) due to susceptibility to the lung pathogen. Plant sterols, particularly β-sitosterol and its glucosides, have been investigated as immune regulators of T-cell activity[30] and as agents in maintaining the CD4^+^ count in the absence of anti-retroviral therapy in HIV-infected patients.[31] Plant sterols were effective in patients treated for pulmonary TB, causing an increase in their peripheral blood lymphocytes and eosinophil counts.[32]

To date, this is the first report on the compounds **2** and **3** from the genus *Alpinia*. More importantly, this article cites for the first time the isolation of all compounds from **A. purpurata**. Furthermore, this study demonstrates the promise of this plant as a source of phytomedicinals that can fight TB.

## References

[1] Chopra I, Khajuria B, Chopra C (1957). Antibacterial properties of volatile principles from *Alpinia galanga* and *Acorus calamus*. Antibiot Chemother.

[2] Lee S, Shin H, Hwang H, Kim J (2003). Antioxidant activity of extracts from *Alpinia katsumadai* seed. Phytother Res.

[3] Madulid DA (1995). A Pictorial Cyclopedia of Philippine Ornamental Plants. Manila: Domingo A. Madulid and Bookmark, Inc.

[4] Ali M, Banskota A, Tezuka Y, Saiki I, Kadota S (2001). Antiproliferative activity of diarylheptanoids from the seeds of *Alpinia blepharocalyx*. Biol Pharm Bull.

[5] Zoghbi M, Andrade E, Maia JG (1999). Volatile constituents from the leaves and flowers of *A. speciosa K. Schum*. and **A. purpurata** (Vieill.) K. Schum. Flavour Fragrance J.

[6] Shimoda H, Shan S, Tanaka J, Okada T, Murai H (2007). Anti-inflammatory agents from red ginger.

[7] Shimoda H (2008). Plant materials for bone and joint diseases: Citrus unshiu and red ginger. .Food Style 21.

[8] Aguinaldo AM (2007). Selected Zingiberaceae species exhibiting inhibitory activity against *Mycobacterium tuberculosis* H_37_ Rv: A phytochemical profile. Gardens’ Bull Singapore.

[9] Mandap K, Marcelo R, Macabeo AP, Yamauchi T, Abe F, Franzblau SG (2007). Phenyldecanoids from the antitubercular fractions of the Philippine ginger (*Zingiber officinale*). ACGC Chem Res Comm.

[10] Collins L LA, F0 ranzblau SG (1997). Microplate Alamar Blue Assay versus BACTEC 460 System for high-throughput screening of compounds against *Mycobacterium tuberculosis* and *Mycobacterium avium*. Antimicrob Agents Chemother.

[11] Kanya, Sindhu TC, Jao JL, Sastry SM (2007). Characterization of wax esters, free fatty alcohols and free fatty acids of crude wax from sunflower seed oil refineries. Food Chem.

[12] Guevara B (2004). A guidebook to plant screening: Phytochemical and biological.

[13] Harbourne JB, Baxter H (1993). Phytochemical dictionary: A handbook of bioactive compounds from plants.

[14] Martin T, Fabon C, Hernandez H (1988). Ultraviolet spectral analysis of flavonoids. Proceedings of the Sub-Regional Workshop on Plant Glycosides I. U.P.

[15] Wang Y, Hamburger M, Gueho J, Hostettman K (1989). Antimicrobial flavonoids from *Psidia trinervia* and their methylated and acylated derivatives. Phytochemistry.

[16] Urbatsch L, Mabry T, Miyakado M, Ohno N, Yoshioka H (1976). Flavonol methyl ethers from *Ericameria diffusa*. Phytochemistry.

[17] Kimura Y, Takido M, Takahashi S (1967). Studies on the constituents of the seeds of *Alpinia japonica*. Yakugaku Zasshi.

[18] Kimura Y, Takido M, Takahashi S, Kimishima M (1967). Studies on the constituents of the seeds of *Alpinia kumatake*. Yakugaku Zasshi.

[19] Pei-Wu G, Fukuyama Y, Rei W, Jinxian B, Nakagawa K (1988). An acylated sitosterol glucoside from Alisma plantago-aquatica. Phytochemistry.

[20] Gomes D, Alegrio L (1998). Acyl steryl glycosides from *Pithecellobium cauliflorium*. Phytochemistry.

[21] Shaiq Ali M, Saleem M, Ahmad W, Parvez M, Raghav Y (2002). A chlorinated monoterpene ketone, acylated β-sitosterol glycosides and flavanone glycoside from *Mentha longifolia* (Lamiaceae). Phytochemistry.

[22] Ahmad V, Aliya R, Perveen S, Shameel M (1992). A sterol glycoside from marine green alga *Codium iyengarii*. Phytochemistry.

[23] Ahmed W, Ahmad Z, Malik A (1992). Stigmasteryl galactoside from *Rhynchosia minima*. Phytochemistry.

[24] Crouch NR, Langlois A, Mulholland DA (2007). Bufadienolides from the southern African Drimia depressa (Hyacinthaceae: Urgineoideae). Phytochemistry.

[25] Ayimele GA, Tane P, Connolly J (2004). Aulacocarpin AB, nerolidol and β-sitosterol glucoside from *Aframomum escapum*. Biochem Syst Ecol.

[26] Kubo I, Muroi H, Himejima H, Kubo A (1993). Antibacterial activity of long-chain alcohols: The role of hydrophobic alkyl groups. Bioorg Med Chem Lett.

[27] Togashi N, Shiraishi A, Nishizaka M, Matsuoka K, Endo K, Hamashima H (2007). *Antibacterial activity* of long chain fatty alcohols against *Staphylococcus aureus*. Molecules.

[28] Murillo J, Encarnacion-Dimayuga R, Malstrom J, Christophersen C, Franzblau SG (2003). Antimycobacterial flavones from *Haploppapus sonorensis*. Fitoterapia.

[29] Fukai T, Sakagami H, Toguchi M, Takayama F, Iwakura I, Atsumi T (2000). Cytotoxic activity of low molecular weight polyphenols against human oral tumor cell lines. Anticancer Res.

[30] Bouic PJ, Etsebeth S, Liebenberg RW, Albrech CF, Pegel K, Van Jaarsveld PP (1996). Beta-sitosterol and beta-sitosterol glucoside stimulate human peripheral blood lymphocyte proliferation: Implications for their use as an immunomodulatory vitamin combination. Int J Immunopharmaco.

[31] Breytenbach U, Clark A, Lamprecht J, Bouic P (2001). Flow cytometric analysis of the Th1-Th2 balance in healthy individuals and patients infected with human immunodeficiency virus (HIV) receiving a plant sterol/sterolin mixture. Cell Biol Int.

[32] Donald PR, Lamprecht JH, Freestone M, Albrecht CF, Bouic PJ, Kotze D (1997). A randomized placebo-controlled trial of the efficacy of beta-sitosterol and its glucoside as adjuvants in the treatment of pulmonary tuberculosis. Int J Tuberc Lung Dis.

